# Ethyl Pyruvate Prevents Methyglyoxal-Induced Retinal Vascular Injury in Rats

**DOI:** 10.1155/2013/460820

**Published:** 2013-02-28

**Authors:** Junghyun Kim, Yun Mi Lee, Chan-Sik Kim, Eunjin Sohn, Kyuhyung Jo, So Dam Shin, Jin Sook Kim

**Affiliations:** Korean Medicine Based Herbal Drug Research Group, Herbal Medicine Research Division, Korea Institute of Oriental Medicine, 1672 Yuseongdaero, Yuseong-gu, Daejeon 305-811, Republic of Korea

## Abstract

Pyruvate is an endogenous antioxidant substance. The aim of this study was to investigate the protective effects of ethyl pyruvate (EP) on retinal vascular injury in diabetic retinopathy. To investigate the protective effect of EP on vascular cell apoptosis and blood-retinal barrier (BRB) breakage, we have used intravitreally methylglyoxal-(MGO-) injected rat eyes. Apoptosis of the retinal vascular cell that was stimulated by the intravitreal injection of MGO was evidently attenuated by the EP treatment. EP exerts inhibitory effect on MGO-induced vascular cell apoptosis by blocking oxidative injury. In addition, EP treatment prevented MGO-induced BRB breakage and the degradation of occludin, an important tight junction protein. These observations suggest that EP acts through an antioxidant mechanism to protect against oxidative stress-induced apoptosis in retinal vessels.

## 1. Introduction

 Diabetic retinopathy is one of the major complications of diabetic mellitus, and the main cause of acquired blindness in working-age adults. Retinal vascular injury, a hallmark of diabetic retinopathy, leads to the retinal pathological changes including thickening of the basement membrane, cellular capillary formation, retinal hemorrhage, endothelial proliferation, and angiogenesis, which ultimately leads to blindness [1–3]. Several studies demonstrated that retinal vascular cell apoptosis plays a crucial role in the development of diabetic retinopathy [4, 5].

 Methylglyoxal (MGO) has been proposed for a causative factor of retinal vascular injury [6, 7]. MGO, a glucose-derived dicarbonyl intermediate, is found in high levels in blood or tissue of diabetic animals and patients [8, 9]. MGO is more reactive than the parent sugars with respect to their abilities to cross-link with amino groups of various protein, forming stable end products called advanced glycation end products (AGEs) [10]. High levels of MGO affect cellular function by reacting with cellular proteins and nucleic acids [11]. There are reports suggesting that the cytotoxicity of MGO is due to its ability to induce apoptosis via oxidative stress [6, 12]. MGO can increase oxidative stress by modifying proteins associated with the formation of reactive oxygen species (ROS) and producing ROS during the glycation reaction [13–15].

Ethyl pyruvate (EP), a stable and lipophilic derivative of pyruvate, is considered to be an effective precursor of pyruvate [16]. The beneficial effects of EP as a potent inflammatory inhibitor and reactive oxygen species scavenger have been estimated in a variety of experimental animal models such as acute pancreatitis [17] and hepatic tumor [18]. EP has also shown renoprotective effect in streptozotocin-induced diabetic rats [19]. Despite the various effects of EP, knowledge of its mechanism of action and the effect on diabetic retinopathy is limited. The current study was conducted to investigate whether EP treatment was able to protect against MGO-induced retinal vascular injury and explore the underlying mechanisms in the intravitreally MGO-injected rat eyes.

## 2. Materials and Methods

### 2.1. Animals and Experimental Design

Thirty-two male SD rats (9 weeks old) were used in this study. Each rat was anesthetized with a 1 : 1 mixture xylazine hydrochloride (4 mg/kg) and ketamine hydrochloride (10 mg/kg). Sixteen rats were injected with a single dose of 6 mM MGO in a volume 4 *μ*L into the vitreous of the right eye with a microinjector (Hamilton Co., NV, USA) under a dissecting microscope. For normal control, 3 *μ*L physiological saline was injected into the left eye. Sixteen rats were preinjected with 2 *μ*L EP (300 mM) for 1 h, followed by 2 *μ*L MGO (12 mM) injection into the right eye. For negative control, 4 *μ*L EP (150 mM) was injected into the left eye. Assuming the vitreous volume of an adult rat eye to be approximately 56 *μ*L [20], the final intravitreal concentration of MGO and EP were approximately 400 *μ*M and 10 mM, respectively. The dose of EP selected for this study was based upon a concentration of EP previously optimized in previous in vitro studies [16]. The needle was left in position for 30–60 s and then slowly withdrawn to minimize fluid loss from the eye. Rats were monitored regularly for infection associated with the injection site. Eyes with injection-damaged lenses or retinas were excluded from the study. At 2 days after the intravitreal injection, rats were anesthetized and killed. All experiments were approved by the Korea Institute of Oriental Medicine Institutional Animal Care and Use Committee. 

### 2.2. Fluorescein-Dextran Microscopy

At necropsy, 1 mL of phosphate buffered saline (PBS) containing 50 mg of fluorescein-dextran (Sigma, St. Louis, MO, USA) was injected into the left ventricle under deep anesthesia. The tracer was allowed to circulate for 10 min and eye was then enucleated and immediately fixed in 4% paraformaldehyde for 2 hours. The plasma was collected and assayed for fluorescence with a spectrofluorophotometer (Synergy™ HT, Bio-Tek, VT, USA) based on standard curves of FITC-dextran in normal plasma. The retinas were dissected, flat mounted onto a glass slide, and viewed by fluorescence microscopy (BX51, Olympus, Tokyo, Japan). Quantification of the fluorescence intensity was calculated by ImageJ software (NIH, MD, USA) and normalized to the plasma fluorescence intensity for each animal.

### 2.3. Preparation of Trypsin-Digested Vessels

The eyes were enucleated from the animals, and the retinas were isolated. After fixation 10% formalin for 2 days, the retinas were incubated in trypsin (3% in sodium phosphate buffer containing 0.1 M sodium fluoride to inhibit the DNase activity) for approximately 60 min. The vessel structures were isolated from the retinal cells by gentle rinsing in distilled water. The vascular specimens were then mounted on slides.

### 2.4. Assessment of Apoptosis

Apoptosis was assessed using a TUNEL staining protocol according to the manufacturer's instructions (Promega, WI, USA). The number of TUNEL-positive cells per unit area (mm^2^) was then determined in and counted in a total of 5 fields. The numbers of apoptotic and total cells were counted. 

### 2.5. Immunofluorescence Staining

The trypsin digests were immunofluorescently stained as previously described [18]. The slides were incubated with a mouse anti-8-hydroxyguanine (8-OHdG) antibody (Santa Cruz, CA, SUA) and a mouse antioccludin antibody (Invitrogen, CA, USA) for 1 h. To detect 8-OHdG and occludin, the slides were incubated with a rodamine-conjugated goat anti-mouse antibody (Santa Cruz). The oxidation of guanine to form 8-OHdG acts as a marker of oxidative DNA damage [19]. The number of 8-OHdG-positive cells was counted per five randomly selected mm^2^ of capillary area using ImageJ software (NIH).

### 2.6. Statistical Analysis

The results were evaluated statistically using one-way analysis of variance followed by Tukey's multiple comparison test using GraphPad Prism 4.0 (GraphPad Softwere, CA, USA). 

## 3. Results

### 3.1. EP Prevented Apoptosis of Retinal Microvascular Cells

**Figure 1:**
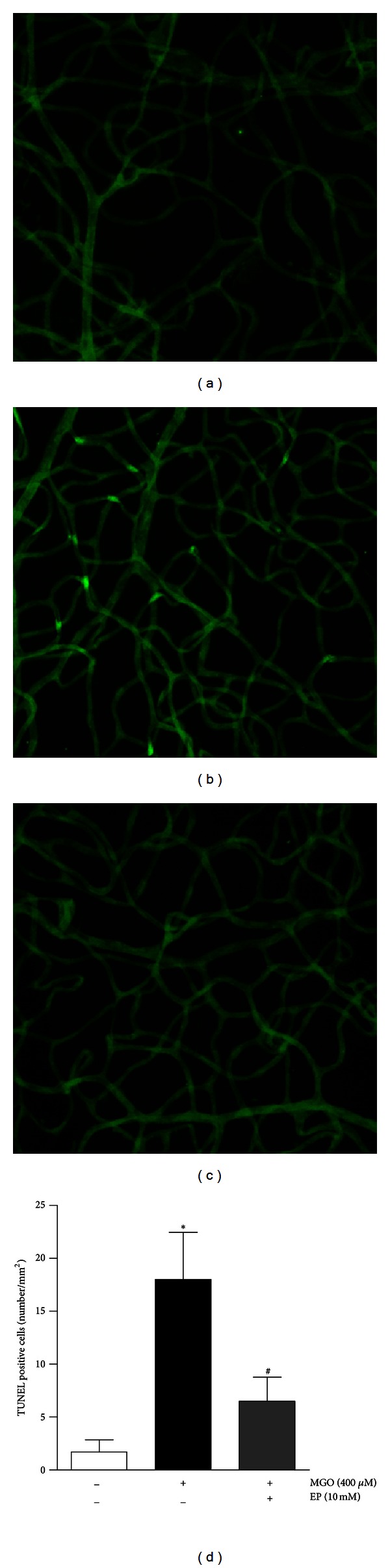
Retinal vascular cell apoptosis in intravitreally MGO-injected rat eyes. The trypsin-digested retinal vessels from a saline-injected eye (a), a MGO-injected eye (b), and an EP-treated eye (c) were stained with TUNEL (green). (d) Quantitative analysis of the TUNEL-positive cells. The values in the bar graphs represent the means ± SE, *n* = 8. _ _*P < 0.01 versus the control group, _ _^#^ *P* < 0.01 versus the MGO-injected group.

To characterize injury of retinal vascular pericyte and endothelial cell by MGO, TUNEL staining was performed using trypsin-digested retinal vessels. TUNEL analysis can detect cells in which DNA is fragmenting and is therefore widely used as a marker for apoptosis [21]. In the retinal trypsin digests of the saline-injected eyes, a TUNEL-positive nucleus was rarely detected (Figure 1(a)). In the MGO-injected eyes, many TUNEL-positive microvascular cells and fragmented nuclei were observed (Figure 1(b)). However, treatment of the MGO-injected eyes with EP prevented the increase in the positive cells that was seen in MGO-injected eyes (Figures 1(c) and 1(d)).

### 3.2. EP Inhibited Oxidative DNA Damage in Retinal Microvascular Cells

**Figure 2:**
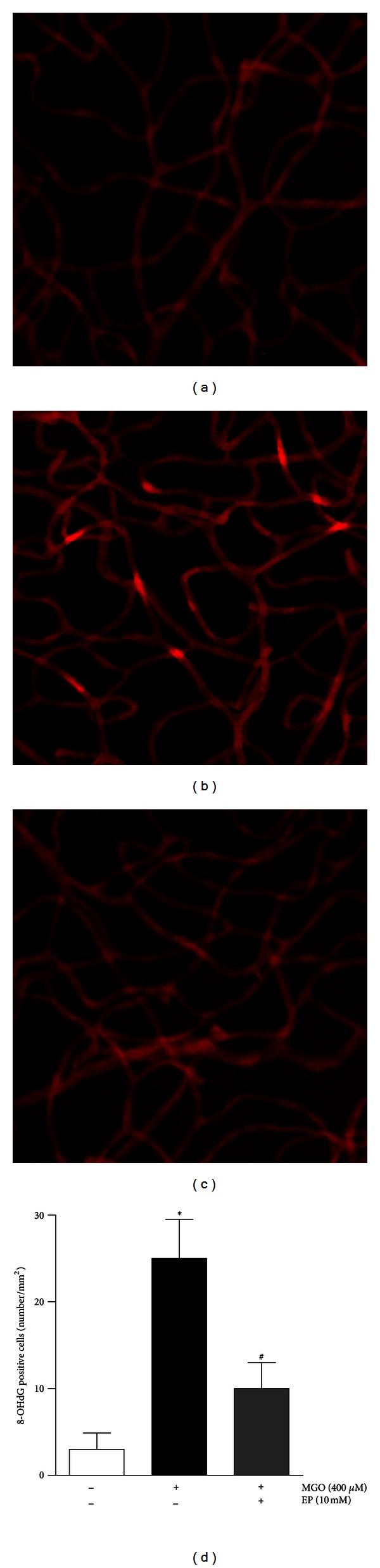
Oxidative DNA damage in retinal vessels derived from intravitreally MGO-injected rat eyes. The retinal tissues were stained with 8-OHdG, which is a marker for oxidative DNA damage (red). Representative photomicrographs of the retinal vasculature from a saline-injected eye (a), a MGO-injected eye (b), and an EP-treated eye (c). (d) Quantitative analysis of the 8-OHdG-positive cells. The data are expressed as the means ± SE. (*n* = 8). _ _*P < 0.01 versus the control group, _ _^#^ *P* < 0.01 versus the MGO-injected group.

Representative patterns of the immunohistochemical localization of 8-OHdG in the trypsin-digested retinal vessels are shown in Figure 2. The formation of 8-OHdG-adducted bases is an oxidative modification of DNA and is considered as a deteriorative consequence of oxidative stress [22]. 8-OHdG marker shows nuclear and/or perinuclear localization in retinal vascular pericytes and endothelial cells. Increased immunoreactivity of 8-OHdG was observed in the MGO-injected eyes. However, the treatment with EP suppressed the expression of 8-OHdG compared to the MGO-injected eyes (Figure 2(d)).

### 3.3. EP Inhibited Blood-Retinal Barrier Breakage

**Figure 3:**
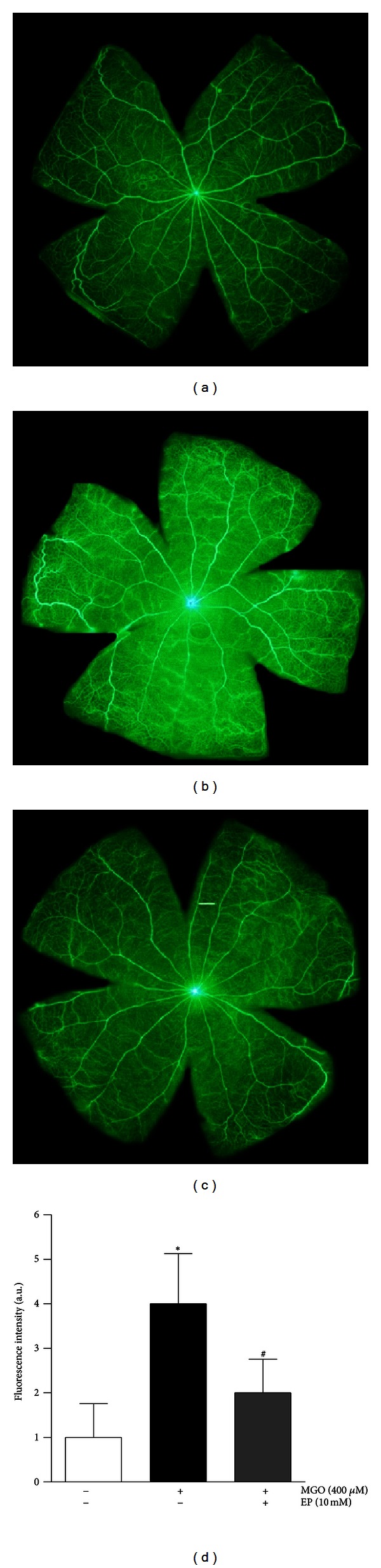
Blood-retinal barrier breakdown. Retinal permeability was determined by the FITC-dextran technique. Representative photomicrographs of the retina from a saline-injected eye (a), a MGO-injected eye (b), and an EP-treated eye (c). Control retina showed no leakage of the tracer into the retina as evidenced by the clear delineation of retinal capillaries. MGO-injected eye demonstrated a widespread breakdown of their BRB with tracer leakage into the neural retina and a loss of delineation of the retinal capillaries. However, the treatment of EP significantly decreased retinal vascular permeability. (d) Quantitative analysis of the immunofluorescence intensity for FITC-dextran. The data are expressed as the means ± SE. (*n* = 8). _ _*P < 0.01 versus the control group, _ _^#^ *P* < 0.01 versus the MGO-injected group.

A major clinical hallmark of diabetic retinopathy is increased capillary permeability culminating in an overt breakdown of the inner blood-retinal barrier (BRB) [21, 23]. In our previous study, MGO disrupts the tight junction protein, leading to breakage of the BRB [24]. EP was tested for its ability to inhibit BRB breakage in retinas. To evaluate increased vascular permeability in MGO-injected eyes, fluorescein angiography was performed using FTIC-dextran.  Figure 3 shows representative fluorescence micrographs of FITC-dextran in control and MGO-injected eyes. The fluorescence intensity diffusely increased throughout the retinal parenchyma in the MGO-injected eyes but is limited to the vasculature in the control and EP-treated eyes.  Figure 3(d) shows the change in the fluorescence intensity in the retinas after normalizing to plasma fluorescence. MGO increased retinal fluorescence by 75% (*P* < 0.01). However, the treatment with EP significantly decreased the fluorescence intensity in the MGO-injected eyes (*P* < 0.01).

### 3.4. EP Prevented Tight Junction Protein Loss

**Figure 4:**
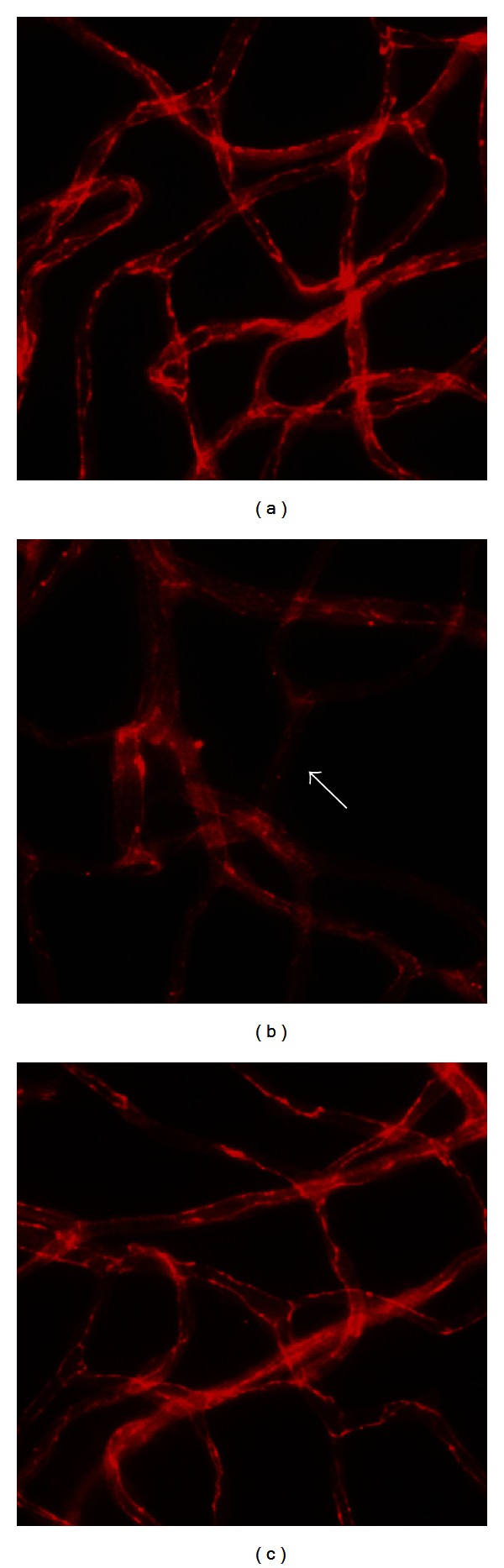
MGO-induced occludin loss. Representative retinal vessels from a saline-injected eye (a), a MGO-injected eye (b), and an EP-treated eye (c) were stained with antioccludin antibody. Occludin expression was evident at the interfaces between adjacent endothelial cells in the control eyes, while it was mostly eliminated from the microvessels in the MGO-injected eyes (arrow).

The loosening of the tight junctions increases retinal vascular permeability [25]. We investigated the expression of tight junction protein known as occludin. As shown in Figure 4, a marked decrease in occludin was detected in the MGO-injected eyes (white arrow). However, treatment of the MGO-injected eyes with EP prevented the loss of occludin in the MGO-injected eyes.

## 4. Discussion

Several studies demonstrated that retinal microvascular cell apoptosis and BRB breakage play a crucial role in the development of early diabetic retinopathy [4, 5]. In the present study, in order to verify the therapeutic effects of EP in diabetic retinopathy, we investigated whether EP prevented this retinal vascular injury in MGO-injected eyes.

Pyruvate is the conjugate anion of pyruvic acid. Pyruvic acid is the final product of glycolysis and the initial substrate for the tricarboxylic acid cycle. The effects of pyruvate on scavenging reactive oxygen radicals have been well known. It became of clinical interest, since reactive oxygen species have been implicated in various injury models. However, the pharmacological application of pyruvate is limited because it is not stable in solution and spontaneously forms potentially toxic metabolites. However, ethyl pyruvate, a derivate of pyruvic acid, is stable in solution and nontoxic [26]. Ethyl pyruvate has demonstrated protective effects in pancreatitis [27], hemorrhagic shock [28] and H_2_O_2_-induced renal injury [29] as well as diabetic nephropathy [19] and liver injury [30]. In addition, there is evidence that ethyl pyruvate can ameliorate vascular injury by inhibiting ROS production and downregulating inflammatory mediators [31, 32].

 Several studies have been reported that the cell death induced by MGO appeared to be due to apoptosis in a variety of cell types [12, 33, 34]. Recently, it was reported that MGO induced the apoptosis of bovine retinal pericytes [6]. In our previous study, MGO is a cause of apoptosis of cultured rat retinal pericytes [35]. MGO has been reported to induce apoptotic cell death via various mechanisms, such as ROS generation [36], p38 MAPK activation [34], JNK pathway [12], and alteration of PDGFR signaling [37]. Recently, it was reported that MGO induced the apoptosis of bovine retinal pericytes by oxidative stress [6]. The treatment with the ROS scavenger, *N*-acetyl cysteine, attenuated MGO-induced apoptotic cell death in rat retinal pericyte [35]. Moreover, in diabetic rats, a mixture of several antioxidants [4] or trolox [38] was able to prevent pericyte loss in retina. To elucidate the mechanism involved in the antiapoptotic effect of EP in rat retinal microvascular cells, we focused on MGO-induced oxidative stress. In the present study, EP inhibited the increase in oxidative DNA damage induced by MGO. When DNA is damaged, cells initiate a cell cycle delay or induction of apoptosis [39]. Diabetes-induced oxidative stress has been well documented in patients and animals [40–42]. Formation of AGEs has been shown to be a source of oxidative stress in retinal endothelial cells via the interaction with AGE receptor [43]. Our results indicate that EP is capable of preventing MGO-induced oxidative stress by virtue of its ability to act as an ROS scavenger.

The destruction of retinal vascular pericytes and endothelial cells results in the formation of acellular capillaries, which are associated with areas of nonperfusion. The increasing of acellular capillaries then leads to the development of retinal ischemia and retinal neovascularization. Therefore, inhibition of apoptosis by EP could be expected to inhibit the development of retinal ischemia and neovascularization. Furthermore, the fluorescein angiography showed that EP markedly inhibited the fluorescein leakage, which suggests that EP might prevent the breakdown of BRB. MGO has been shown to induce ROS generation in the endothelium [44]. Oxidative stress is known for its deleterious effects, which can cause enhanced permeability of the BRB [45, 46]. The retinal endothelial monolayer exhibits high transendothelial resistance, a property that is attributed to intercellular tight junctions between apposed endothelial cells [47]. Among the several tight-junctional proteins, occludin is pivotal in the regulation of endothelial monolayer electrical resistance, paracellular solute permeability, and normal barrier function [48]. Although occludin by itself cannot form a functionally tight barrier, it likely plays an important role in the organization and stabilization of the tight junction. MGO-induced tight-junctional disruption could be a mechanism in barrier impairment associated with vascular pathology, such as in diabetes [9, 49]. Therefore, MGO-mediated disruption of occludin is expected to increase endothelial permeability, and the attenuation of MGO stress would preserve barrier integrity. Indeed, our result showed that MGO-induced occludin degradation was attenuated by the treatment of EP. The results suggest that EP also has potential to prevent BRB dysfunction induced by MGO.

 In conclusion, our study shows that EP inhibits the retinal vascular cell apoptosis and BRB breakage induced by MGO in rats. In addition, oxidative stress induced by MGO was significantly inhibited by treatment with EP. The attenuation of MGO stress by EP preserved the tight junction protein occludin integrity. These observations suggest that EP acts through an antioxidant mechanism to protect against oxidative stress-induced retinal vascular injury. Taken together, these results indicate that treatment with EP could be a valuable therapeutic approach in the treatment or prevention of diabetic vascular injury.
